# Editorial: Cannabis and cannabinoids in psychiatry

**DOI:** 10.3389/fpubh.2025.1618252

**Published:** 2025-05-13

**Authors:** Pooja R. Sarkar, Mark S. Gold, Kevin P. Hill

**Affiliations:** ^1^Beth Israel Deaconess Medical Center, Boston, MA, United States; ^2^Department of Psychiatry, Harvard Medical School, Boston, MA, United States; ^3^Department of Psychiatry, School of Medicine, Washington University in St. Louis, St. Louis, MO, United States

**Keywords:** cannabis (marijuana), tetrahydrocannabinol (THC), cannabis and psychosis, cannabis and health behavior, cannabis use disorder

Cannabis, often marketed as a promising therapeutic tool, is also associated with adverse effects including psychosis, cognitive deficits and risk of cannabis use disorder (1). As commercial interests expand, so too does the need for further research on cannabis' impact on health and functioning. From exploring perceived benefits, to studying associated risks, the articles in this Research Topic identify trends in public impact and delineate future exploratory paths. In this Research Topic, authors also consider the ever-changing nature of product potency, and physician attitudes around cannabis as a potential treatment in their own practice.

The growing popularity of higher-tetrahydrocannabinol (THC) potency products and low perceived harm create a perfect storm for elevated risk of adverse effects (2, 3). Individuals consuming high-THC cannabis daily are more prone to early-onset psychosis, suggesting that there is a dose-response relationship between THC potency and adverse mental health outcomes (1, 4, 5). In this article Research Topic, Elsohly et al. build on prior work that reviewed two decades of Δ^9^-THC and cannabidiol (CBD) concentrations in samples seized by the Drug Enforcement Administration (DEA) (6, 7). This latest study re-characterizes regional cannabis samples to identify potential chemical variations in the type or potency of cannabis used. Regional variation in potency was hypothesized, particularly as markets and legal access to cannabis vary by state and products cannot be legally transported between states. The chemical compositions of cannabis were found to be relatively homogenous across the regions and similar to in structure to medical cannabis from dispensaries in states with legalized access. These findings have significant public health implications as the general comparability of cannabis chemical profiles and THC potency enable more uniform and broadly applicable interventions and policy.

In addition to characterizing potency trends, understanding the impact of cannabis on functioning and productivity is an important feature of public health. The intersection of cannabis use and behavior is an under-explored part of public health, particularly in the context of health-promoting behaviors (8, 9). YorkWilliams et al. explore the association between physical exercise, a health-promoting behavior, and cannabis use before and/or after exercise (co-use) in states with full legal access. They explored aerobic and anaerobic exercise behavior in individuals using cannabis, and found that those who co-use cannabis demonstrate, on average, significantly greater total weekly duration of exercise than their counterparts who do not use cannabis immediately before or after exercise. This investigation lays the groundwork to better understand how the perception of cannabis' effect on exercise can impact the health-promoting behavior itself. Future endeavors in this area should study exercise behaviors of individuals who are completely abstinent from cannabis, as cannabis use overall may influence exercise behaviors. Similarly, this work focuses on states where cannabis has been legalized, and, as aptly noted by the authors, there may be further observations gleaned from states where cannabis use is not legal.

Analyzing positive attitudes and perceived benefits of cannabis use is an worthwhile endeavor, as is considering adverse associations that impact functioning. Déguilhem et al. analyzed individual-level data on cannabis use frequency and sick leave from a French national cohort (CONSTANCES cohort) of over 220,000 adult volunteers in metropolitan France, who were covered by the National Health Insurance Fund from 2012 to 2018. Data was linked with the National Health Data System (NHDS), which also provided detailed information about dates and durations of sick leave. Cannabis used more than monthly was associated with increased risk of short sickness absences (< 7 days), even when controlled for age and gender. This frequency of cannabis use was also associated with significantly increased risk of medium sickness absences medium (7 to 28 days) in older and male participants. This dose-dependent association lays the foundation for further study of the economic and public health impact of cannabis.

Taken together with data on increased workplaces risks, including an increased risk of falls and decreased alertness with cannabis use (10), these findings suggest that there is an observable association between workplace absenteeism and cannabis use that warrants further research on the risk cannabis use may introduce into the workplace. Distilling our understanding of the association between cannabis and adverse health effects remains an important undertaking, particularly as cannabis use impacts the prognosis of mood disorders, such as major depressive disorder (MDD) and bipolar disorder (BD) which are highly co-morbid with cannabis use disorder (11).

Sorkhou et al. review evaluates longitudinal and cross-sectional studies on the association between cannabis use and mood disorders. They found cannabis use was not only associated with worsened depressive and manic symptoms, but also with increased suicidality and risk of developing major depressive and bipolar disorders. These findings help identify areas for future study, including exploration of individuals who use cannabis but do not have an MDD or BD diagnosis. These findings from Sorkhou et al. underscore the need to interrogate claims made by medical cannabis proponents that there are therapeutic benefits that outweigh associated risks.

Elucidating cannabis' therapeutic potential is further complicated by variable medical opinions amongst physicians. In this Research Topic, Syed et al. query the experience and attitudes of physicians on the therapeutic role of medical cannabis and furthermore evaluate comfort with prescribing medical cannabis in their own practice. By evaluating an array of international perspectives, this work identifies domain-specific knowledge gaps about medical cannabis use, and highlights notable variability in physician willingness to prescribe. The heterogenous comfort levels with prescribing medical cannabis deviate from the current landscape of evidence, which is considered inconclusive at best. As such, this work is an important next step to understanding how physician knowledge on the topic may impact practice.

The science of cannabis is still in its early stages, while the marketing and public acceptance of cannabis is further along. Cannabis products are only increasing in variety and popularity, and thus the study of its market evolution, associated risks, and perceived benefits must also diversify.
